# Metabolite Damage and Damage Control in a Minimal Genome

**DOI:** 10.1128/mbio.01630-22

**Published:** 2022-07-11

**Authors:** Drago Haas, Antje M. Thamm, Jiayi Sun, Lili Huang, Lijie Sun, Guillaume A. W. Beaudoin, Kim S. Wise, Claudia Lerma-Ortiz, Steven D. Bruner, Marian Breuer, Zaida Luthey-Schulten, Jiusheng Lin, Mark A. Wilson, Greg Brown, Alexander F. Yakunin, Inna Kurilyak, Jacob Folz, Oliver Fiehn, John I. Glass, Andrew D. Hanson, Christopher S. Henry, Valérie de Crécy-Lagard

**Affiliations:** a Department of Microbiology and Cell Science, University of Florida, Gainesville, Florida, USA; b Horticultural Sciences Department, University of Florida, Gainesville, Florida, USA; c Food Science and Human Nutrition Department, University of Florida, Gainesville, Florida, USA; d J. Craig Venter Institute, La Jolla, California, USA; e Chemistry Department, University of Florida, Gainesville, Florida, USA; f Maastricht Centre for Systems Biology (MaCSBio), Maastricht University, Maastricht, The Netherlands; g Department of Chemistry, University of Illinois at Urbana-Champaign, Urbana, Illinois, USA; h Department of Biochemistry, University of Nebraska, Lincoln, Nebraska, USA; i Department of Chemical Engineering and Applied Chemistry, University of Toronto, Toronto, Canada; j Centre for Environmental Biotechnology, School of Natural Sciences, Bangor University, Bangor, United Kingdom; k West Coast Metabolomics Center, UC Davis, Davis, California, USA; l Data Science and Learning, Argonne National Laboratory, Argonne, Illinois, USA; m Consortium for Advanced Science and Engineering, The University of Chicago, Chicago, Illinois, USA; n University of Florida Genetics Institute, Gainesville, Florida, USA; o Redox Biology Center, University of Nebraska, Lincoln, Nebraska, USA; University of Arizona

**Keywords:** comparative genomics, metabolite repair, metabolomics, minimal genome, hydrolase

## Abstract

Analysis of the genes retained in the minimized *Mycoplasma* JCVI-Syn3A genome established that systems that repair or preempt metabolite damage are essential to life. Several genes known to have such functions were identified and experimentally validated, including 5-formyltetrahydrofolate cycloligase, coenzyme A (CoA) disulfide reductase, and certain hydrolases. Furthermore, we discovered that an enigmatic YqeK hydrolase domain fused to NadD has a novel proofreading function in NAD synthesis and could double as a MutT-like sanitizing enzyme for the nucleotide pool. Finally, we combined metabolomics and cheminformatics approaches to extend the core metabolic map of JCVI-Syn3A to include promiscuous enzymatic reactions and spontaneous side reactions. This extension revealed that several key metabolite damage control systems remain to be identified in JCVI-Syn3A, such as that for methylglyoxal.

## INTRODUCTION

A foundational goal of synthetic biology was to create a minimal living organism by a bottom-up approach (1). This goal was reached in 2016 with the creation of JCVI-Syn3.0 (2). This organism was built from the ruminant pathogen Mycoplasma mycoides subsp. *capri* serovar LC GM12 by DNA synthesis, recombination, and genome transplantation techniques and included only genes required for survival or to support a reasonable growth rate (428 protein-coding genes and 34 RNA genes) (2). The initial JCVI-Syn3.0 strain was extremely fragile; a derivative with 18 more genes, JCVI-Syn3A, was more stable and was the basis for a metabolic model (3). Surprisingly, when JCVI-Syn3.0 was published in 2016, ~30% of its genes could not be assigned a specific function. The initial annotation has since been improved by manual curation (4), metabolic modeling (3), and further *in silico* analyses (5), but ~85 proteins with unknown or vaguely defined functions remain (see Supplemental data A1 at figshare [https://doi.org/10.6084/m9.figshare.20020574]). These unknowns cannot all be missing parts of synthesis/breakdown pathways as the metabolic reconstruction identified only four metabolic and eight transport reactions as missing (3).

A crucial area of metabolism usually left out of metabolic models is metabolite damage and repair. Enzymes make mistakes, and metabolites undergo spontaneous chemical reactions (6, 7). These damage reactions are ever present and, when the resulting products are toxic, can reduce fitness (6, 8). It has been shown recently that many enzymes of formerly unknown function repair or preempt metabolite damage (9–11), that mutations in metabolite repair enzymes cause human diseases (12–14), and that pathway engineering can fail unless appropriate repair enzymes are installed (15). The emerging recognition of the nature and extent of metabolite damage and repair raised the question of the importance of metabolite repair for a minimal genome like JCVI-Syn3/3A. By combining expert manual curation, comparative genomics, metabolomics, metabolic modeling, cheminformatics, and experimental validation, we identified a set of chemical damage reactions likely to occur in JCVI-Syn3 and some of the damage repair and preemption activities that this minimal genome encodes.

## RESULTS AND DISCUSSION

### Identification and validation of homologs of known metabolite repair enzymes.

We first manually screened the predicted proteome of JCVI-Syn3A for homologs of known metabolite repair enzymes (6, 15, 16) (see Supplemental data S1 and Appendix at figshare [https://doi.org/10.6084/m9.figshare.20020574]). Several were found, as follows.

### (i) 5-FCL.

**FIG 1:**
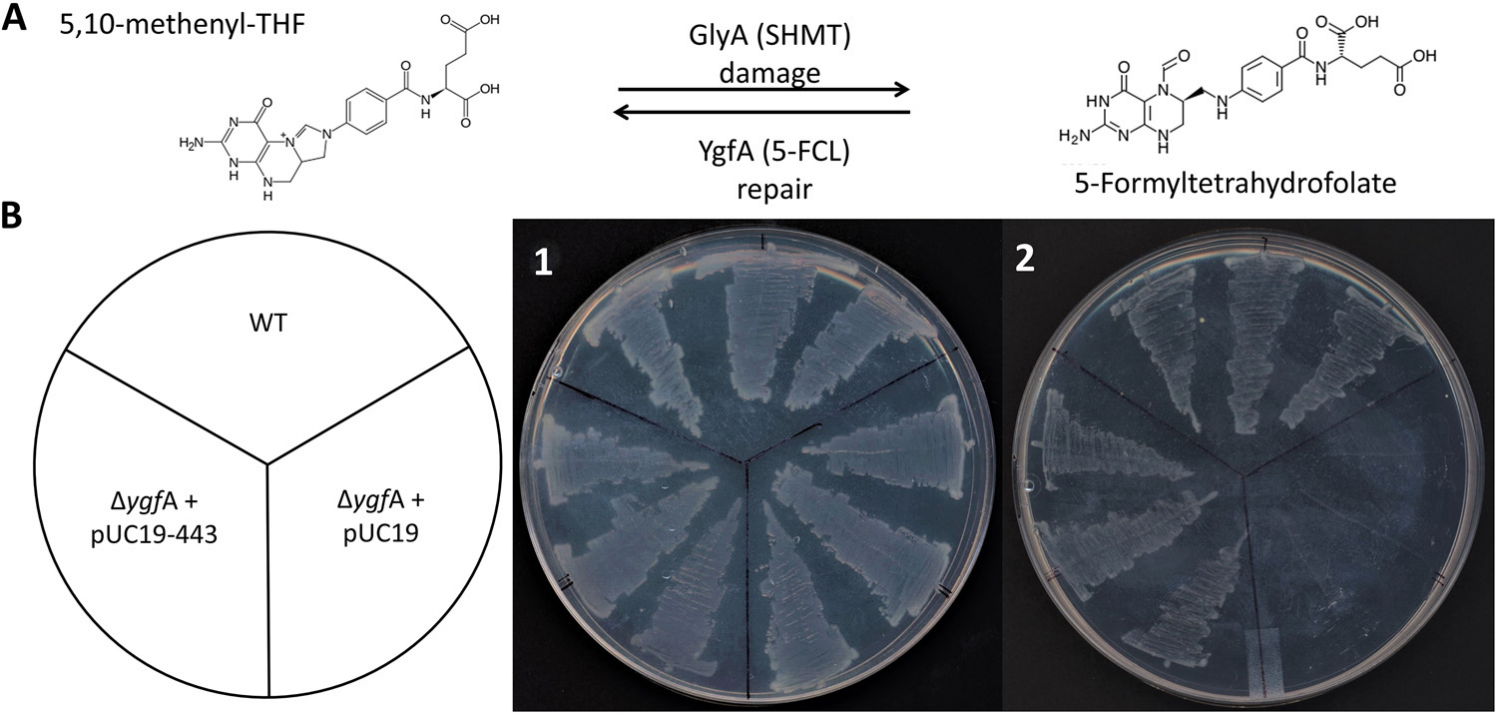
5-FCL activity is encoded by JCVISYN3A_0400. (A) Enzymatic source and repair of 5-CHO-THF. (B) Growth phenotype of a wild-type (WT) E. coli BW25113, a Δ*ygfA* mutant, and a Δ*ygfA* mutant expressing the JCVISYN3A_0443 gene on M9 minimal medium (0.4% glucose) with 20 mM NH_4_Cl (plate 1) or 50 mM glycine (plate 2) as sole nitrogen source. Plates were incubated for 3 days at 37°C.

5-Formyltetrahydrofolate (5-CHO-THF) is a by-product of serine hydroxymethyltransferase (SHMT) (17) (Fig. 1A) that inhibits folate-dependent enzymes and must therefore be recycled or destroyed (18). Of various enzymes known to recycle 5-CHO-THF (19), the most widespread is 5-formyltetrahydrofolate cycloligase (5-FCL) (encoded by *fau/ygfA* [16] in Escherichia coli). The JCVI-Syn3A genome encodes a 5-FCL homolog (JCVISYN3A_0443); this gene was confirmed to encode an active 5-FCL by a complementation assay. Specifically, an E. coli K-12 Δ*ygfA* strain does not grow on M9 minimal medium with 0.2% glucose as carbon source and 20 mM glycine as sole nitrogen source (19) (Fig. 1B). Expression of JCVISYN3A_0443 from a plasmid complemented this growth phenotype (Fig. 1B). Note that the essentiality of JCVISYN3A_0443 might be due both to its repair function and to a role as a source of 5,10-methenyltetrahydrofolate-polyglutamate (3).

### (ii) Thiol reductases.

**FIG 2:**
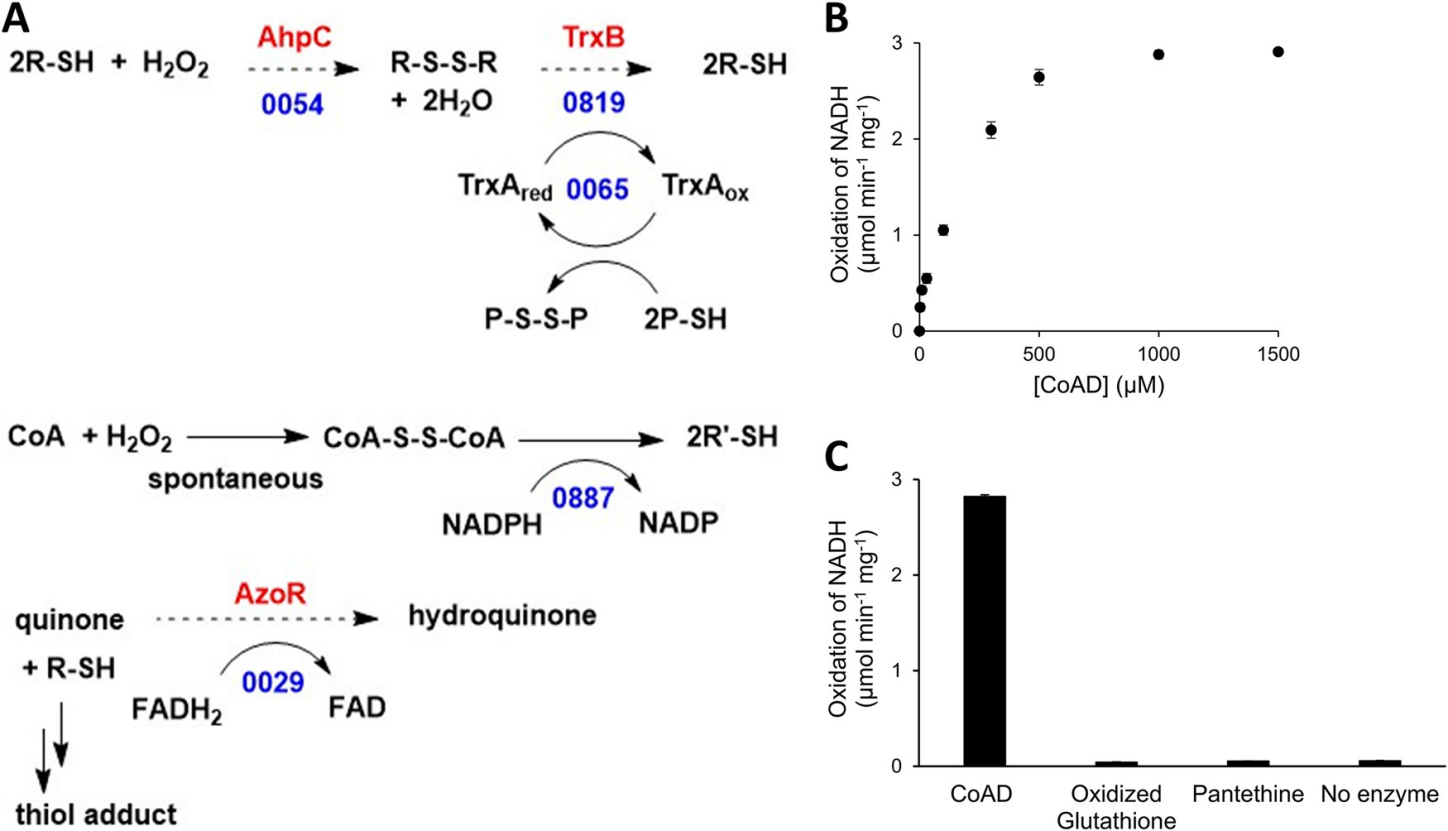
Predicted and validated redox buffering systems in JCVI-Syn3. (A) Candidates for H_2_O_2_ detoxification systems of JCVI-Syn3; those experimentally validated are shown by solid arrows, only the numbers of the locus tags are given, P is for protein, and R is for small molecule. The predicted source of reductant is NADPH. (B) CoADR Michaelis-Menten saturation curve for the determination of the *K_m_* and *k*_cat_ for CoAD consumption. (C) CoADR is specific toward oxidized CoA with no activity toward other tested disulfides.

Like all aerobes, JCVI-Syn3A encounters oxidative stress that can damage macromolecules. Maintaining protein and small-molecule thiol groups in their reduced state is critical for cellular redox homeostasis (20). Thioredoxin/thioredoxin reductase is the dominant protein thiol oxidoreductase system in many organisms, using reducing equivalents ultimately derived from NAPDH (21, 22). The JCVI-Syn3A genome encodes homologs of the thioredoxin system proteins (TrxB/JCVISYN3A_0819 and TrxA/JCVISYN3A_0065) that are most likely involved in reducing protein disulfide bonds and have been partially characterized in other *Mycoplasma* species (Fig. 2A) (23, 24). Both genes are essential (see Supplemental data A1 at figshare [https://doi.org/10.6084/m9.figshare.20020574]), supporting key roles for TrxA and TrxB in disulfide bond reduction. Note, however, that thioredoxin is also the electron donor for ribonucleotide reductase, so that JCVISYN3A_0819 and JCVISYN3A_0065 may be essential for this reason (23, 25).

JCVISYN3A_0887 is a homolog of coenzyme A (CoA) disulfide reductase (CoADR), which may have a major redox role in certain bacteria (26). Because CoA is required for several reactions in the JCVI-Syn3A metabolic model and is predicted to be imported from the medium, CoADR could maintain the CoA pool in the reduced state. Testing the CoADR activity of JCVISYN3A_0887 showed that it is an active CoADR that operates well at physiological pH (pH 7.5) (27) and has reasonable *K_m_* (0.17 mM) and *k*_cat_ (2.8 s^−1^) values (Fig. 2B). It lacks detectable activity against oxidized glutathione or pantethine (Fig. 2C). While we cannot exclude the possibility that reduced glutathione is imported from the medium and oxidized glutathione is exported, a CoA-based system is a more parsimonious solution to the redox balance problem.

### Functional analysis of HAD proteins identifies a nucleotide phosphatase with possible dual roles.

Our second strategy to identify metabolite repair enzymes was based on the demonstration that hydrolases of previously uncertain or unknown function were subsequently shown to participate in metabolite repair (9). Five genes encoding standalone members of the HAD (haloacid dehalogenase) hydrolase family (28) were identified in the JCVI-Syn3A genome (see Supplemental data S1 at figshare [https://doi.org/10.6084/m9.figshare.20020574]) and are conserved in the closely related Mesoplasma florum L1 genome (29) (Table 1). Such HAD hydrolases often participate in metabolite repair or homeostasis, as many damaged or toxic intermediates are phosphorylated (e.g., phosphosugars), and their recycling or removal requires a phosphatase (9, 30).

**TABLE 1:** Members of the HAD family of unknown function encoded by JCVI-Syn3

Gene	Family	Essential	Best 3 substrates with activity *in vitro*^*a*^	Physical clustering	*M. florum* ortholog, locus tag and essentiality^*b*^
JCVISYN3A_0066	Cof subfamily of IIB subfamily of HAD superfamily	No	*p*NPP, FMN, CoA	Between 5S rRNA gene and thioredoxin	Mfl169 (NE)
JCVISYN3A_0077	Cof-like hydrolase, HAD superfamily	No	Fru-1P, Ery-4P	Between *tsaD* and *aspS*	Mfl614 (E)
JCVISYN3A_0710	Cof subfamily of IIB subfamily of HAD superfamily	Yes	Could not clone	Between tRNA genes and predicted phosphonate transporter genes	Mfl513 (E)
JCVISYN3A_0728	HAD superfamily hydrolase subfamily IIB, protein	No	GMP; XMP; 2-deoxyglucose-6P	Between glycolysis genes	Mfl503 (E)
JCVISYN3A_0907	Cof-like hydrolase, HAD superfamily	No	*N*-Acetyl-d-glucosamine-6P; fructose-1P; *N*-acetyl-d-glucosamine-1P	Between YidC and choline kinase-like	Mfl680 (NE)

a Abbreviations are in Table S1 at figshare (https://doi.org/10.6084/m9.figshare.20020574).

b E, essential, and NE, nonessential in *M. florum*.

Comparative genomic analysis of the standalone HADs did not point to clear functional hypotheses, except for JCVISYN3A_0728, whose location in a predicted operon with triose-phosphate isomerase and phosphoglycerate mutase suggested a role in sugar phosphate metabolism (Table 1). Possible functions for the HAD proteins included (i) repair of substrates to be identified, (ii) missing phosphatases involved in primary metabolism identified by the metabolic model such as sedoheptulose 1,7-bisphosphate phosphatase or phosphatidate phosphatase, and (iii) nucleotide phosphatases involved in deoxynucleoside triphosphate (dNTP) pool maintenance. To discriminate among these hypotheses, we combined biochemistry, genetics, and metabolomics.

The four HAD proteins that we were able to express in E. coli (JCVISYN3A_0066, JCVISYN3A_0077, JCVISYN3A_0728, and JCVISYN3A_0907) were tested for activity against a panel of 94 phosphatase substrates (see Table A1 at figshare [https://doi.org/10.6084/m9.figshare.20020574]) (31). The four proteins had detectable activity against the model phosphatase substrate *p*-nitrophenyl phosphate (*p*NPP) and different physiological substrates (see Fig. A1 at figshare [https://doi.org/10.6084/m9.figshare.20020574]). The JCVISYN3A_0728 enzyme hydrolyzed a wide range of nucleoside and sugar phosphates, the JCVISYN3A_0907 and JCVISYN3A_0077 enzymes hydrolyzed narrower ranges of sugar phosphates, and the JCVISYN3A_0066 enzyme hydrolyzed flavin mononucleotide (FMN) and CoA. That sugar phosphates are good substrates of JCVISYN3A_0728 is consistent with its genomically predicted role in sugar phosphate metabolism, but no specific function or substrate could be assigned. Note, however, that the 94-substrate panel did not include damaged sugar phosphates.

We attempted to delete HAD-encoding genes in JCVI-Syn3A, expecting this to be possible because transposon bombardment of the JCVI-Syn3A genome indicated that all five HADs were quasiessential (i.e., required for fast growth but not for viability) (3) (see also Supplemental data S1 at figshare [https://doi.org/10.6084/m9.figshare.20020574]). Deletants were readily obtained for genes JCVISYN3A_0066, JCVISYN3A_0077, JCVISYN3A_0728, and JCVISYN3A_0907 (see Supplemental data S2 at figshare [https://doi.org/10.6084/m9.figshare.20020574]). Attempts to delete JCVISYN3A_0710 using two different methods were unsuccessful (see Supplemental data S2 at the figshare URL above). Deletion of this gene could have resulted in an extremely slow-growing strain that was unrecoverable under the conditions used, Alternatively, JCVISVN3A_0710 could be essential, the transposon insertions in the gene being artifacts. That the same gene is also essential in *M. florum* (Table 1) favors the latter hypothesis.

We observed no major differences in growth rates between JCVI-Syn3A and any HAD mutant (see Fig. A2 at figshare [https://doi.org/10.6084/m9.figshare.20020574]). To conduct a metabolomics analysis, the four mutants and the JCVI-Syn3A parent were grown in SP4-KO medium (defined in Materials and Methods) and harvested at the same point of log-phase growth. (see the Appendix and Tables A2 and A3, all at figshare [https://doi.org/10.6084/m9.figshare.20020574]). A total of 4,152 features were detected in the samples using hydrophilic interaction liquid chromatography (HILIC) and mass spectrometry (see Supplemental data S3 at figshare [https://doi.org/10.6084/m9.figshare.20020574]), of which 522 were annotated as known metabolites.

**FIG 3:**
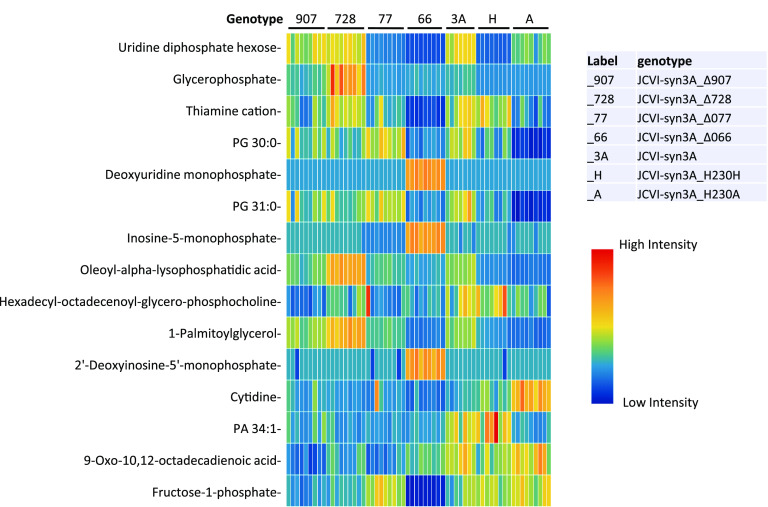
Heatmap including 15 metabolites from JCVI-Syn3A mutant metabolomic analysis with highest VIP scores. Samples and genotypes are represented in columns. High-intensity measurements compared to average intensity are red/yellow, and low-intensity measurements are represented by green/blue.

Partial least-squares discriminant analysis was used to find the variable importance in projection (VIP) scores of each annotated metabolite. The 15 metabolites with the highest VIP scores (Fig. 3; see also Fig. A3 at figshare [https://doi.org/10.6084/m9.figshare.20020574]) showed little contamination from media, as determined by analysis of unused media along with mutant samples. Most of these metabolites were below the limit of detection in unused media, and most of the rest were present at a >30-fold-lower abundance in media than in samples, suggesting little or no contamination from residual media (see Supplemental data S3 at figshare [https://doi.org/10.6084/m9.figshare.20020574]). Two metabolites (cytidine and thiamine) were found at similar abundances in media and samples, suggesting these medium contaminations.

Among the 15 metabolites with high VIP scores, the JCVISYN3A_0728 knockout showed significantly higher abundances of glycerophosphate, oleoyl lysophosphatidic acid, and palmitoylglycerol than other genotypes (Fig. 3; see also Fig. A3 at figshare [https://doi.org/10.6084/m9.figshare.20020574]). We were not able to determine which form of glycerophosphate was increased, although the 3-phosphate is *a priori* more likely, being found in the metabolic model as a cardiolipin metabolism intermediate that is synthesized via phosphorylation of imported glycerol by GlpK (JCVISYN3A_0218). To further analyze the knockout metabolomics data further, all four HAD hydrolase knockout phenotypes were separately compared to wild-type JCVI-Syn3A (see Supplemental data S3 at figshare [https://doi.org/10.6084/m9.figshare.20020574]). The conclusions are summarized below and further discussed in the Appendix at figshare (https://doi.org/10.6084/m9.figshare.20020574).

The metabolomics data suggest that JCVISYN3A_0066 is the major deoxyribonucleoside monophosphatase (dNMPase) with activity against the deoxymononucleotides dAMP, dGMP, dUMP, dCMP, dTMP, and dIMP and also the ribomononucleotide IMP. Furthermore, as further discussed in the Appendix at figshare (https://doi.org/10.6084/m9.figshare.20020574), the data also suggest the residual presence of pyrimidine nucleoside phosphorylase (PyNP) activity in JCVI-Syn3A after the known MMSYN1_0734 has been removed. The lack of observed nucleotidase activity for JCVISYN3A_0066 in the *in vitro* substrate screen could be due to the absence of relevant effectors. In contrast, it seems that JCVISYN3A_0077 is also a dUMP-specific specific dNMPase that plays a minor role *in vivo* compared to JCVISYN3A_0066. The metabolomics data also suggest that JCVISYN3A_0728 is a glycerol 3-phosphate phosphatase. The other activities detected *in vitro*, if relevant *in vivo*, might not be apparent in the metabolomics data if these substrates do not accumulate in cells. No functional role could be proposed for JCVISYN3A_0907.

### Comparative genomics uncovers a possible metabolite repair diphosphatase.

**FIG 4:**
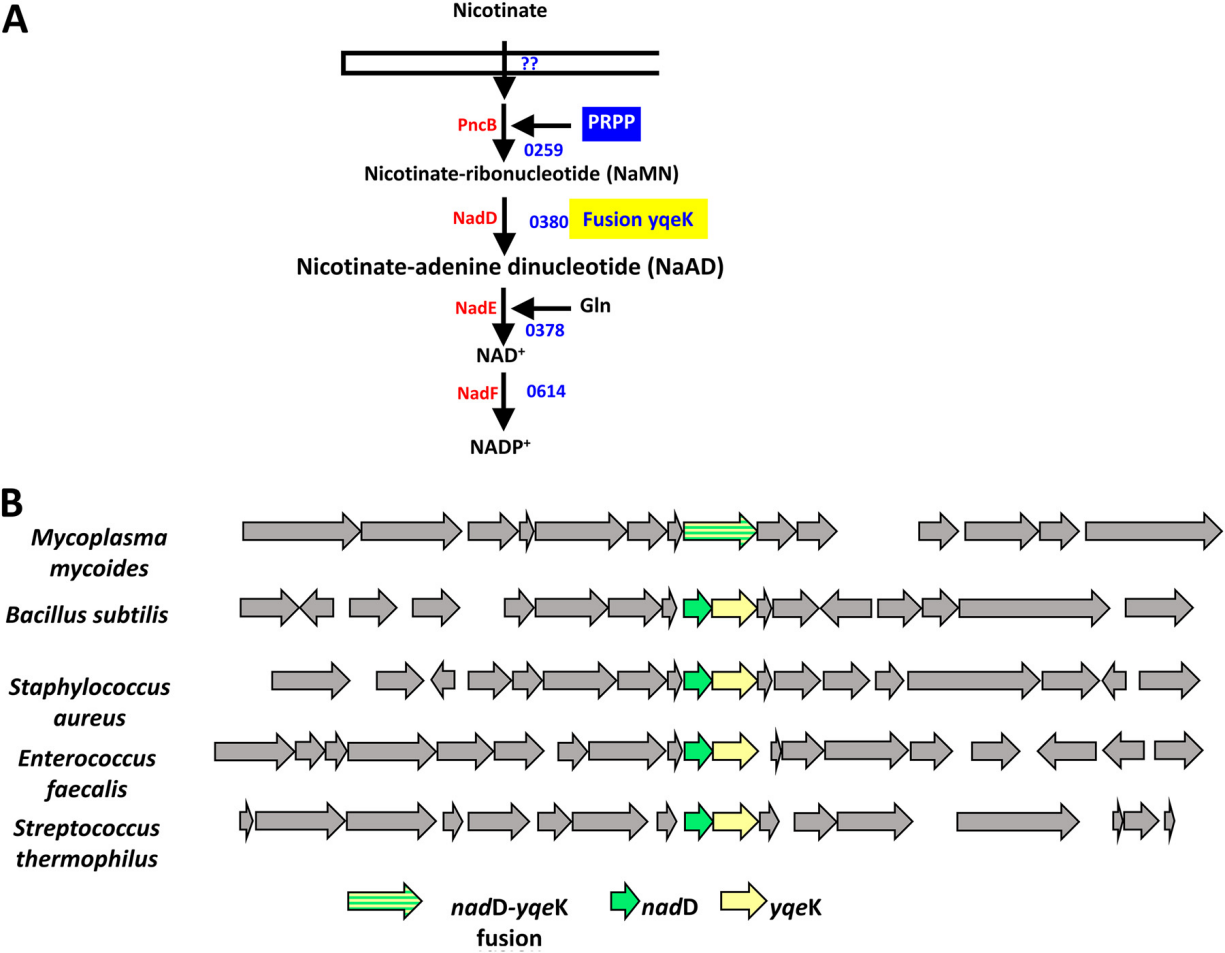
Predicted hydrolase of unknown function is clustered or fused to NadD in many *Firmicutes*. (A) Predicted NADP^+^ synthesis pathway in JCVI-Syn3. (B) Physical clustering and fusions of *nadD* and *ykeK* homologs in several Gram-positive bacteria. The RefSeq identifiers for the *yqeK* genes used in descending order are CAE77070, NP_390441.1, by AAW38265.1, AAO82560.1, and AAV61221.1.

**FIG 5:**
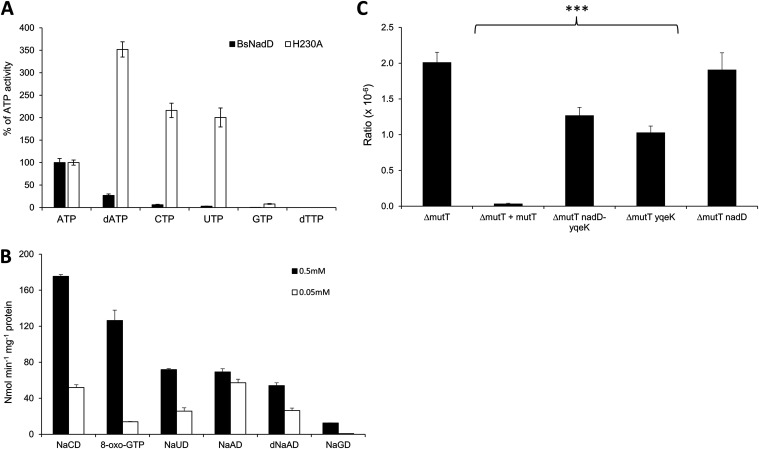
Biochemical analysis of the NadD and YqeK activities. (A) Relative reaction rates of Bacillus subtilis and JCVI-Syn3.0 NadD enzymes with NaMN and various nucleotides, calculated as percentage of the canonical reaction with ATP for each NadD enzyme. Enzymes were incubated with 2 mM NTP, 0.5 mM NaMN, 4 mM MgCl_2_, and 5 U/mL yeast inorganic pyrophosphatase for 5 min at 37°C. H230A has the conserved H in the active site of the YqeK domain mutated to ablate the HD activity and cleavage of nucleotides. (B) Activity of the expressed JCVI-Syn3.0 YqeK domain with different substrates. YqeK (0.2 μg) was incubated with 0.5 or 0.05 mM concentrations of the substrates, 1 mg/mL bovine serum albumin, and 2.0 mM MgCl_2_ for 20 min at 37°C. Black bars are data for 0.5 mM substrates, and white bars are data for 0.05 mM substrates. (C) Mutation ratio on LB-rifampicin for Δ*mutT* strain with empty vector (pBAD24), Δ*mutT* strain with E. coli *mutT* in *trans*, and Δ*mutT* strain with either the *nadD*-*yqeK* fusion gene JCVISYN3A_0380 or the *nadD* or *yqeK* domain alone. *** indicates a *P* value of <0.001 with experiments performed with four biological replicates and four technical replicates.

The YqeK histidine -aspartate (HD)-domain phosphohydrolase is fused to nicotinic acid mononucleotide adenylyltransferase (NadD) in most mycoplasmas and strongly physically clustered with NadD in many other Gram-positive organisms (32) (Fig. 4; see also Fig. A4A at figshare [https://doi.org/10.6084/m9.figshare.20020574]). These genomic associations led us to propose that YqeK repairs mistakes made by NadD. The canonical activity of NadD is to adenylate nicotinate-ribonucleotide (NaMN) using ATP as a donor of the AMP moiety (Fig. 4A). However, use of another NTP or the deoxy-form of ATP would create an erroneous product requiring disposal, most likely by hydrolysis. We therefore expressed JCVISYN3A_0380 and its His230Ala variant in E. coli (see Fig. A4B at figshare [https://doi.org/10.6084/m9.figshare.20020574]). (The His230Ala mutation is predicted to abolish phosphatase activity that would interfere with NadD activity measurement.) Bacillus subtilis NadD was used as a benchmark. The JCVISYN3A_0380 His230Ala protein and B. subtilis NadD were tested for *in vitro* activity with various nucleoside triphosphates as the substrates. The adenylating activity of the JCVISYN3A_0380 His230Ala mutant was quite nonspecific and actually greater against dATP, CTP, or UTP than against the physiological substrate, ATP, whereas B. subtilis NadD strongly preferred ATP (Fig. 5A). JCVI-Syn3 NadD can therefore readily form deoxyadenosine, deoxycytidine, or deoxyuridine analogs of the NAD precursor nicotinate adenine dinucleotide (NaAD), which can presumably be converted to inhibitory analogs of NAD and NADP.

We then tested the JCVI-Syn3 YqeK domain for diphosphatase activity using the NaAD analogs that could be produced by JCVI-Syn3A NadD. The YqeK domain had activity toward the cytosine (NaCD) and uracil (NaUD) analogs of NaAD that was at least as high as that against NaAD itself (Fig. 5B), which agrees with the preference of the NadD domain to form these analogs.

We also observed that the YqeK domain had high activity against 8-oxo-GTP, although judging from relative activities with 0.05 mM and 0.5 mM substrate, the *K_m_* is likely higher than that for the other substrates tested (Fig. 5B). Consistent with this finding, we showed that the genes encoding the JCVI-Syn3A NadD-YqeK fusion can partially complement the E. coli *mutT* high-mutation-rate phenotype (measured as Rif^r^ ratios) (Fig. 5C). The partial complementation was also observed when expressing the YqeK domain alone but not the NadD domain alone. Finally, it was recently shown that YqeKs of Gram-positive bacteria belong to a novel diadenosine tetraphosphate (Ap4A) hydrolase family (33). Taken together, these observations suggest that YqeK is a versatile diphosphatase with several functional roles.

Indeed, the available transposon insertion data (3) (see also Supplemental data S1 at figshare [https://doi.org/10.6084/m9.figshare.20020574]) suggested that the NadD domain is essential and the YqeK domain is quasiessential because a few hits in the YqeK region of the gene were detected in the first Transposon (Tn) round and disappeared after the fourth round. We could not isolate a JCVISYN3A_0380 deletant despite several attempts. We were, however, able to construct a strain carrying the His230Ala mutation that inactivates YqeK diphosphatase activity (see Supplemental data S2 at figshare [https://doi.org/10.6084/m9.figshare.20020574]), and this strain showed no growth defect or obvious metabolite imbalance (see Fig. A2 at figshare [https://doi.org/10.6084/m9.figshare.20020574]).

### Metabolomics-driven exploration of damage and repair chemistry in JCVI-Syn3.

Thus far, all of our damage and repair cases began with analysis of genes in the JCVI-Syn3A genome and uncovered clear instances of metabolite damage and repair. But are these examples isolated exceptions, or the tip of an iceberg of uncharacterized metabolic chemistry? To address this question, we adopted a systematic exploratory approach based on the metabolomics data for JCVI-Syn3A cells (see Table S3 at figshare [https://doi.org/10.6084/m9.figshare.20020574]). Because this approach begins with the observed chemical results of potential metabolite damage and is not limited by our current knowledge of gene function, it will certainly find damage mechanisms that our gene-first approach will miss. Still, this approach will also miss any damage mechanisms that fail to be observed through metabolomics, either due to volatility of end products or due to extremely effective damage mitigation systems.

**FIG 6:**
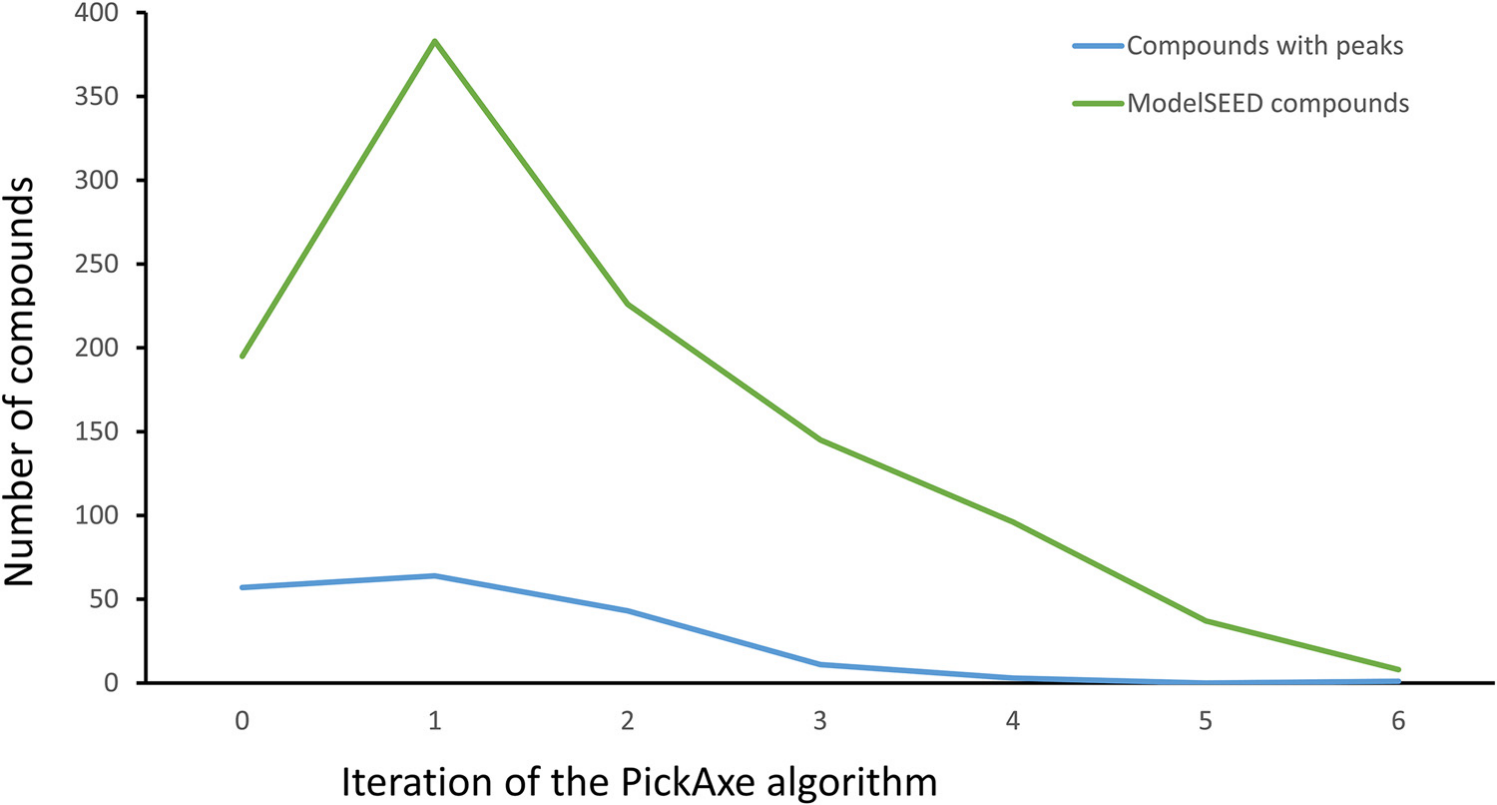
Number of predicted potential metabolites arising from promiscuous enzymatic reactions and spontaneous/damage chemistry operating on known compounds in JCVI-Syn3 metabolism. Total predicted metabolites are shown, as well as the number of metabolites matching observed peaks (blue line) or ModelSEED compounds (green line). The *x* axis indicates the number of reaction steps explored outward from the known JCVI-Syn3 metabolism, while the *y* axis shows the number of new metabolites predicted with each new reaction step.

We focused specifically on a set of 480 metabolites (see Table S4E at figshare [https://doi.org/10.6084/m9.figshare.20020574]) that satisfied two criteria: (i) the mass spectral signal was confidently identified with a defined molecular structure, and (ii) the metabolite was at least as abundant in the JCVI-Syn3A cells as in the growth medium. We compared the 480 identified peaks to the 304 metabolites in the JCVI-Syn3A model and the 33,978 compounds in the ModelSEED database (34), resulting in 57 matches to the model and 217 (45%) matches to the database (see Table S4E at the figshare URL above). The comparison to the JCVI-Syn3A model reveals two types of discrepancy: (i) 247 metabolites in our model do not appear in our metabolomics data, which is to be expected as many metabolites are too low in concentration or too volatile to be detected in metabolomics, and (ii) 423 metabolites that were observed and do not appear in our model, which is more problematic as this implies that there is significant chemistry taking place in this system that our present model cannot explain. The ModelSEED database lookup reveals further discrepancies: (i) 263 observed metabolites do not appear in biochemistry databases, indicating that there is no known biochemical route to any of these compounds that are observed to arise in a biological system, and (ii) 160 observed metabolites (217 to 57) do have known biochemical biosynthesis mechanisms, but these mechanisms do not appear in our current JCVI-Syn3 model (3). To predict potential chemical routes to as many of the observed metabolites as possible without limiting our search to known chemistry or straying too far from known JCVI-Syn3A metabolism, we used the PickAxe tool (35). This tool applies generalized reaction rules based on known spontaneous (8) and enzymatic (36, 37) chemical mechanisms to predict potential novel reactions that a given set of metabolites (here, all JCVI-Syn3A metabolites) could undergo. We started with the 304 metabolites present in the JCVI-Syn3A model and applied PickAxe for multiple iterations to allow generation of multistep pathways (see Materials and Methods). We used both spontaneous and enzymatic reaction rules in the PickAxe expansion, enabling prediction of pathways with a mixture of both (as occurs in many damage and repair pathways). The initial PickAxe iterations uncovered an increasing number of compounds generated that matched the observed metabolites, but these hits tapered off after six iterations to just one new compound produced that matched an observed metabolite (blue line in Fig. 6). The number of compounds predicted by PickAxe that matched known biochemistry in the ModelSEED database (green line in Fig. 6) followed a similar trend. We halted the PickAxe expansion at this stage, given its diminishing returns. The final chemical network generated by PickAxe included 33,934 compounds and 61,939 reactions and matched a total of 182 distinct metabolites (including the original 57 matching the JCVI-Syn3 model) and 1,090 ModelSEED compounds (see Supplemental data S4C and D at figshare [https://doi.org/10.6084/m9.figshare.20020574]).

**FIG 7:**
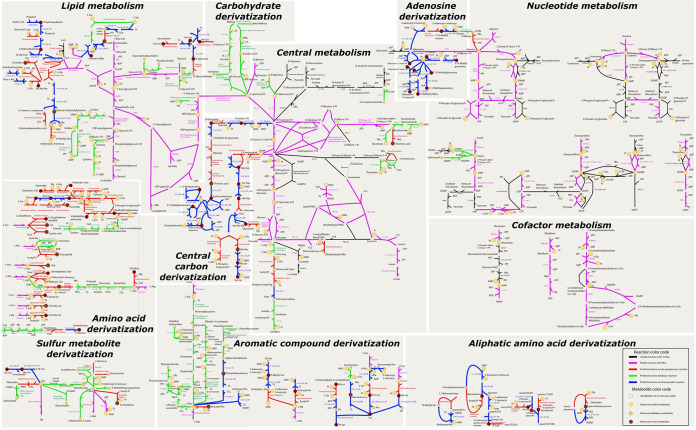
Map of predicted extensions to the JCVI-Syn3 model to push flux through as many observed peaks as possible. Reactions and metabolites are color coded as shown in the inset. Model reactions with no flux are black, and those with flux are magenta. Predicted and active reactions that are in the database are green, those that are novel and spontaneous are red, and those that are novel enzymatic ones are blue. All active predicted spontaneous reactions and nearly all active model reactions are shown on the map; some ModelSEED and predicted enzymatic reactions are excluded. The color code for metabolites is as follows: those absent in the mass spectrometry analysis are white, observed metabolites that are also in the model are yellow, those in the database are ocher, and those that are novel in themselves or in the way they are produced are brown. Most enzymatic reactions are identified by their EC numbers. Some reactants’ names have been omitted since they do not give relevant information. Common abbreviations have been used for the name labels. The map has been divided by panels shown on the figure’s background. These panels are labeled according to the major pathway they display. The complete metabolic map in interactive format (Escher map) is given in Supplemental data S5 at figshare (https://doi.org/10.6084/m9.figshare.20020574).

Next, we used a new flux balance analysis (FBA) formulation, metabo-FBA, to select a minimal subset of these reactions that connect the functioning JCVI-Syn3A model to as many observed metabolites as possible using mass- and energy-balanced pathways (see Materials and Methods). Because our study is of a minimal genome with relatively few enzymes and specifically focuses on metabolite damage, we favored solutions that involved as many reactions generated by spontaneous reaction rules as possible. This approach produced a predicted flux profile that simultaneously pushed flux through reactions involving compounds that matched 182 observed metabolites (see solution depicted in Fig. 7 and data in Supplemental data S4A and E at figshare [https://doi.org/10.6084/m9.figshare.20020574]). This solution included 145 (58%) of the 252 reactions in the JCVI-Syn3 model (purple reactions in Fig. 7), 129 additional ModelSEED reactions (primarily predicted enzymatic reactions; green reactions in Fig. 7), 84 novel enzymatic reactions (blue reactions in Fig. 7), and 74 novel spontaneous reactions (red reactions in Fig. 7) (see data in Supplemental data S4A at the figshare URL above). The fixed image of our flux solution depicted in Fig. 7 is of limited value for permitting a detailed exploration of the fluxes, so we are also including all data files and instructions needed to replicate this view in a fully functioning dynamic Escher map (see Supplemental data S5 at figshare [https://doi.org/10.6084/m9.figshare.20020574]). Also, the fully expanded version of the JCVI-Syn3A model used to generate this flux solution is provided in SBML and JSON format in Supplemental data S5 at the figshare URL above.

This flux solution is only one of many possible solutions that can explain the observed metabolomics data. While it is unlikely that this solution is completely correct, the true solution must make use of similar chemistry, start with the same initial high-confidence JCVI-Syn3A compounds, and produce the same observed metabolic intermediates, meaning the true solution cannot differ very substantially from our selected one.

The map broadly (Fig. 7) shows clear hot spots of chemical expansion (adenine, cytosine, sugars, pyruvate, amino acids, central carbon trunk reactions, and CoA) and regions with little or none (deoxynucleotides, guanine, thymidine, THF, riboflavin, NAD). This is probably explained by the intrinsic reactivities and the concentrations of the associated compounds. Many of the hot spot compounds are high-concentration metabolic starting points (e.g., sugars), end points (e.g., amino acids), or high-flux intermediates (e.g., pyruvate). Their high concentrations make it more likely that these compounds will react chemically and that metabolomics will detect the resulting products.

The many ModelSEED reactions and predicted novel enzymatic reactions proposed by this approach represent previously unannotated but potential promiscuous side activities of existing annotated gene products in JCVI-Syn3A. The metabolomic evidence for the presence of the products of these reactions points strongly to the presence of the reactions themselves. The cluster of ModelSEED reactions expanding from the glucose-6-phosphate (g6p) node of the JCVI-Syn3A model is a good example (Fig. 7). These reactions are phosphorylations and hydrolyses that interconvert many diverse sugars and polysaccharides, all of which are supported by our metabolomics data. As the model contains reactions for glucose only as a representative sugar, it probably understates the extent of such reactions.

Also of note is how many of the pathways predicted in JCVI-Syn3A by our metabo-FBA method involve a mixture of database reactions, predicted spontaneous reactions, and novel enzymatic reactions (30/50 total pathways). Any analysis based on just one or two of these three reaction sources would explain a far smaller number of observed metabolites due to gaps and dead ends in the predicted pathways. Thus, using all three reaction data sources provides a much fuller understanding of metabolism.

Another notable point is that much of the new predicted chemistry surrounds amino acids. Many of the observed metabolomics peaks correspond to amino acid derivatives such as dipeptides and acetylated amino acids (Fig. 7). The dipeptides serve primarily as nutrients for JCVI-Syn3, which contains the peptidases needed to degrade these compounds (a large number of the ModelSEED reactions added by our metabo-FBA approach relate to dipeptide transport and degradation). The acetylated amino acids are different in that only 7 out of 10 of these compounds were found in biochemistry databases, and the databases lacked spontaneous acetylation reactions to produce these compounds. Yet, metabolomics evidence supported the presence of all 10 in the JCVI-Syn3A strain. The metabo-FBA approach added 10 predicted spontaneous acetylation reactions, using acetyl-phosphate as a donor, based on PickAxe predictions. This demonstrates how readily acetylation occurs in these systems, either by spontaneous action or by promiscuous enzyme activity, and it highlights the particular vulnerability of amino acids to this acetylation.

These results also support previous hypotheses about the main metabolic network of JCVI-Syn3A (3) with regard to acetyl phosphate and the enzymes producing/consuming it. The *in vivo* essentiality of phosphate acetyltransferase (JCVISYN3_0229) and acetate kinase (JCVISYN3_0230) was previously puzzling, given that the upstream genes in the pathway, the subunits of pyruvate dehydrogenase (JCVISYN3_0227/8), were found to be nonessential *in vivo*. It had been hypothesized that the two former enzymes were essential because buildups of acetyl-CoA or acetyl phosphate needed to be prevented, both being known protein acetylation agents (38). The extensive and diverse acetylation damage we found evidenced in our metabolomics data would seem to further support this hypothesis.

Relatedly, our results support a role for acetyl phosphate in the acetylation of proteins as well as free amino acids because some of the identified amino acids had side chain acetylations. The results also support the hypothesized essential role of acetate kinase as a means of preventing acetyl phosphate accumulation.

These analyses also expose insights into the relative importance of our various proposed mechanisms for spontaneous chemistry, based on which mechanisms are most likely to give rise to metabolic products found in our metabolomics data (see larger discussion in the Appendix and Fig. A5 at figshare [https://doi.org/10.6084/m9.figshare.20020574]). Of course, not all chemically impactful metabolites are readily observed in metabolomics data due to instability or volatility. Methylglyoxal is a good example of an important metabolite that arises from and participates in spontaneous damage reactions but could not be observed (Fig. 7). While methylglyoxal was not among the observed metabolites due to small size and volatility, metabo-FBA added reactions involving this compound because it leads to numerous downstream potential damage and repair reactions. A more detailed discussion of methylglyoxal follows.

### Possible ways for JCVI-Syn3A to cope with methylglyoxal stress.

Methylglyoxal is necessarily formed from the triose phosphates in JCVI-Syn3A central metabolism (39), but the classical glyoxalase system comprising the glutathione-dependent GloA and GloB enzymes (40) is absent. Likewise, the JCVI-Syn3A genome does not encode enzymes with minor methylglyoxal-detoxifying activities, such as aldose reductases and ketoaldehyde reductases (41–43). The only candidate gene for an enzyme that we identified as potentially able to counter methylglyoxal-induced damage is JCVISYN3A_0400, which encodes a homolog of DJ-1. The DJ-1 superfamily has several functionally distinct clades, of which four are found in E. coli (encoded by *hchA*, *yajL*, *yhbO*, and *elbB*). Phylogenetic analysis places JCVISYN3A_0400 in the YajL/DJ-1 clade (see Fig. A6 at figshare [https://doi.org/10.6084/m9.figshare.20020574]).

**FIG 8:**
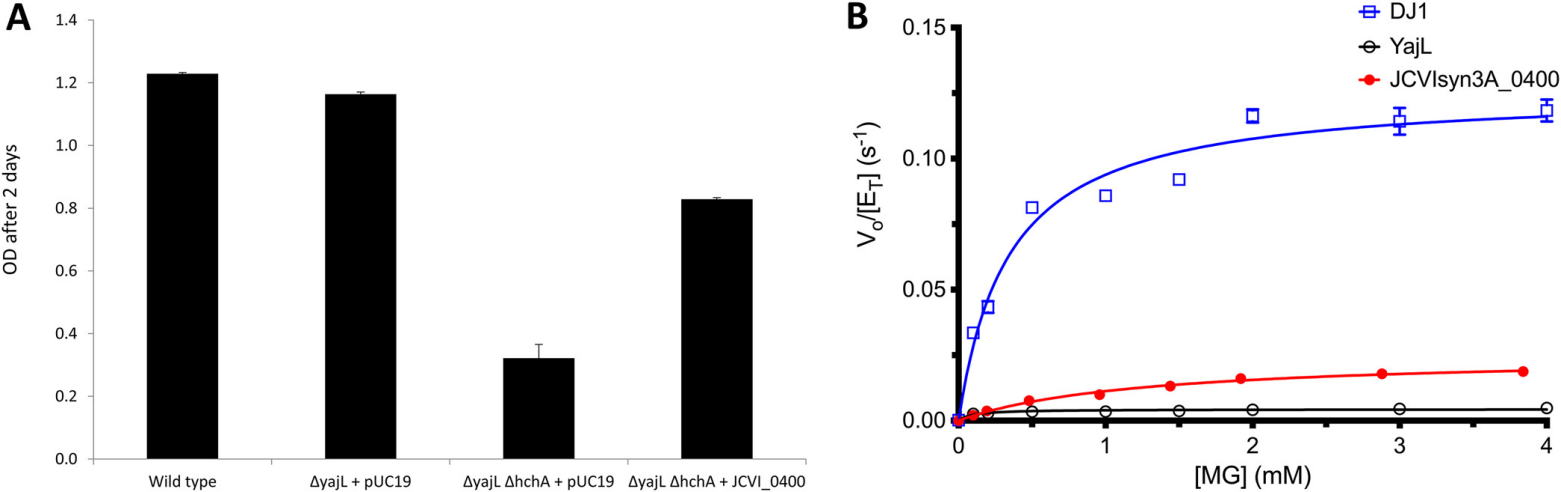
Characterization of JCVISYN3A_0400. (A) Growth of wild-type, Δ*yajL*, and Δ*yajL* Δ*hchA* strains; Δ*yajL* Δ*hchA* strain with *hchA* in *trans*; and Δ*yajL* Δ*hchA* strain with JCVISYN3A_0400 in *trans*. pUC19 was used as an empty vector. Each strain was tested in 5 replicates. Plates were incubated 2 days at 37°C in LB with agitation in a Bioscreen C device. OD, optical density. (B) Methylglyoxalase (MG) activity of JCVISYN3A_0400 compared to human DJ-1 (DJ1) and E. coli YajL. Conversion of methylglyoxal to l-lactate was measured in a coupled assay with l-lactate oxidase and Amplex red. Data were measured in triplicate with error bars shown (sometimes smaller than the symbol) and fitted using the Michaelis-Menten model. JCVISYN3A_0400 is a weak methylglyoxalase.

The members of the DJ-1 superfamily that have been functionally characterized participate in stress response and detoxification (44). Some are thought to be deglycases (45), glyoxalases (46), or aldehyde-adduct hydrolases (47). Previous studies showed variability in the phenotypes reported for the E. coli *hchA*, *yajL*, and *yhbO* deletion mutants as the sensitivity of the *yajL* mutant reported by the Richarme group (48) was not reproduced in independent studies (46). We also failed to reproduce the reported glyoxal or methylglyoxal sensitivities of the single-deletion *yajL* mutant but did observe a defect both in its growth rate and in the yield of the Δ*yajL*/Δ*hchA* E. coli K-12 BW25113 strain (Fig. 8A; see also Fig. S7A at figshare [https://doi.org/10.6084/m9.figshare.20020574]). Expression of the E. coli *yajL* or JCVISYN3A_0400 gene in *trans* complemented this growth phenotype (Fig. 8A; see also Fig. A7A at figshare [https://doi.org/10.6084/m9.figshare.20020574]), suggesting that JCVISYN3A_0400 is indeed in the same DJ-1 subgroup as YajL.

To test the hypothesis that JCVISYN3A_0400 participates in methylglyoxal detoxification, we measured the glyoxalase activity of the recombinant protein. JCVISYN3A_0400 possess a low but measurable methylglyoxalase activity (*k*_cat_ = 0.025 ± 0.002 s^−1^, *K_m_* = 1.23 ± 0.30 mM), lower than that obtained for the positive-control protein human DJ-1 (*k*_cat_ = 0.126 ± 0.004 s^−1^, *K_m_* = 0.34 ± 0.04 mM) but higher than that for E. coli YajL (*k*_cat_ = 0.004 ± 0.0001 s^−1^, *K_m_* = 0.095 ± 0.018 mM) (Fig. 8B). The low *k*_cat_ for YajL is consistent with a prior report that did not detect glyoxalase activity using methylglyoxal as a substrate (46). The ~20 M^−1^ s^−1^ *k*_cat_/*K_m_* value for JCVISYN3A_0400 is 5 to 6 orders of magnitude lower than that of glyoxalase I, the canonical glutathione-dependent glyoxalase (49). Even compared to other DJ-1 superfamily glyoxalases, JCVISYN3A_0400 is a poor enzyme. The lactate oxidase-coupled assay used here is specific to l-lactate, which should detect all the lactate produced by JCVISYN3A_0400, as a prior study indicated that DJ-1 clade enzymes produce only the l enantiomer (50), although we did not test the enantiopurity of the lactate produced by JCVISYN3A_0400 in this study.

Because DJ-1 superfamily members have been reported to be generalist deglycases (51), we tested the deglycase activity of JCVISYN3A_0400 against the methylglyoxal-CoA hemithioacetal (see Fig. A7B at figshare [https://doi.org/10.6084/m9.figshare.20020574]). CoA was used as the thiol because the absence of glutathione biosynthetic enzymes in JCVI-Syn3A suggests that CoA is its main small-molecule thiol (see above). JCVISYN3A_0400 had no detectable deglycase activity against methylglyoxal-CoA hemithioacetal (see Fig. A7B at the figshare URL above), while human DJ-1 had a low activity (*k*_cat_ = 0.0068 ± 0.0007 s^−1^, *K_m_* = 0.144 ± 0.064 mM) against the same substrate (52). JCVISYN3A_0400 therefore seems unlikely to efficiently detoxify methylglyoxal via either glyoxalase or deglycase pathways. It is possible that JCVISYN3A_0400 and other DJ-1-type glutathione-independent methylglyoxalases have some unidentified positive effector *in vivo* that enhances their activity, and the glyoxalase activity of human DJ-1 is highly sensitive to buffer conditions (53). In summary, while results suggest that JCVISYN3A_0400 and YajL are isofunctional, they do not appear to make a large contribution to methylglyoxal detoxification.

The recent observation that human DJ-1, E. coli YajL, and Schizosaccharomyces pombe DJ-1 can reduce the levels of modifications derived from 1,3-bisphosphoglycerate suggests an alternative hypothesis for the function of JCVISYN3A_0400 and other close DJ-1 homologs (54). It is possible that these proteins share an evolutionarily conserved function in detoxifying an electrophilic cyclic 1,3-phosphoglycerate intermediate that is spontaneously formed by intramolecular cyclization of 1,3-bisphosphoglycerate (54). This metabolite should be formed in all organisms that use glycolysis and thus provides a possible explanation for why the minimal *Mycoplasma* JCVI-Syn3A would need to preserve this pathway.

### Conclusion.

Metabolite damage arising from side reactions of enzymes and spontaneous chemistry has often been ignored or seen as a minor metabolic inconvenience—even a trivial sideshow—that does not warrant investment in enzymes to prevent or repair it (6). Biochemical, genetic, and engineering evidence accumulating over the past decade has started to change this view (6, 8, 13, 15, 55, 56). The biochemical and genetic results presented here constitute persuasive additional evidence by demonstrating that stripping a genome down to its barest essentials leaves metabolite damage control systems in place. Furthermore, our metabolomic and cheminformatic results point to the existence of a network of metabolite damage and damage control reactions that extends far beyond the corners of it characterized so far. In sum, there can be little room left to doubt that damage itself and the systems that counter it are mainstream metabolic processes.

## MATERIALS AND METHODS

### Bioinformatics.

The BLAST tools (57) and CDD resources at NCBI (http://www.ncbi.nlm.nih.gov/) (58) were routinely used. Sequences were aligned using Clustal Omega (59) or Multalin (60). Phylogenetic distribution was analyzed in the SEED database (61). Results are available in the “YqeK” subsystem on the PubSEED server (http://pubseed.theseed.org//SubsysEditor.cgi?page=ShowSpreadsheet&subsystem=NadD-YqeK_fusion_display). Physical clustering was analyzed with the SEED subsystem coloring tool or the SeedViewer Compare Regions tool (61), and the clustering figure was generated with Gene Graphics (62). Phylogenetic trees were constructed with MEGA6 (63). Student’s *t* test calculations were performed using the VassarStats web tools (http://vassarstats.net).

### Prediction of novel potential chemistry using PickAxe.

Expanded chemistry was generated using the PickAxe app in KBase, as shown in the narrative at https://narrative.kbase.us/narrative/29280. This app uses the open-source RDKit package to apply sets of SMARTS-based chemical reaction rules, derived from previously published chemical damage (8) and enzyme promiscuity (35) studies, to an input set of compounds to produce all possible reactions and products that might arise from that chemistry. This analysis can be run iteratively through repeated application of the reaction rules to all new products that arise from previous generations. We applied the PickAxe approach for six iterations, retaining all compounds that matched the JCVI-Syn3A model, the ModelSEED database (34), or an observed metabolite.

### Metabo-FBA to predict minimal reactions to reach observed metabolites.

In metabo-flux balance analysis (metabo-FBA), constraints are added to the standard FBA formulation to force flux through one or more reactions involving an observed metabolite. In this formulation, a variable is added for each observed peak (*p_i_*) and a variable is added for each metabolite that has been mapped to the peak (because peaks lack stereochemistry, they may be mapped to multiple possible stereoisomers). Next, a constraint is added stating that a peak cannot be active unless one or more of its associated metabolites is active (where λ*_i_*_,_*_j_* is a mapping variable equal to 1 if metabolite *j* is mapped to peak *i* and zero otherwise):

$$p_{i}\;\leq \;\overset{\text{Compounds}}{\underset{j}{\sum }}{\text{λ}}_{i,j}m_{j}$$

A constraint is also added stating that no metabolite can be active unless at least one reaction in which the metabolite is involved is carrying flux (where γ*_j_*_,_*_k_* is a mapping variable equal to 1 if metabolite *j* is involved in reaction *k* and zero otherwise):

$$m_{j}\;\leq \;\overset{\text{Reactions}}{\underset{k}{\sum }}100{\text{γ}}_{j,k}v_{k}$$

To maximize active metabolites, the objective of the problem is then set to maximize the sum of all *p_i_*. While *p_i_* and *m_j_* can be specified as binary variables, it works equally well and is less computationally expensive to use continuous variables bounded between 0 and 0.1. To avoid the trivial solution of activating metabolites by pushing flux through both directions of reversible reactions or around mass-balanced flux loops, it is essential to also employ thermodynamics constraints in some form in this formulation (64).

### Media, strains, and genetic manipulations.

All strains, plasmids, and oligonucleotides used in this study are listed in Table A4 and Table A5 at figshare (https://doi.org/10.6084/m9.figshare.20020574). Bacterial growth media were solidified with 15 g/L agar (BD Diagnostics Systems) for the preparation of plates. E. coli was routinely grown on LB medium (BD Diagnostics Systems) at 37°C unless otherwise stated. Transformations were performed following standard procedures (62). Isopropyl-β-d-thiogalactopyranoside (IPTG; 100 μM), ampicillin (Amp; 100 μg/mL), kanamycin (Km; 50 μg/mL), l-arabinose (Ara; 0.02 to 0.2%), chloramphenicol (Cm; 25 μg/mL), and rifampicin (Rif; 25 μg/mL) were used when appropriate. Bacterial M9 minimal medium (65), 0.4% (wt/vol) glucose, was used either with NH_4_Cl (20 mM) or with glycine (50 mM) as the nitrogen source. P1 transduction was performed following the classical methods (66). The Kan^r^ marker was eliminated from the BW2113 Δ*yajL*::Kan^r^ strain by the procedure described by Cherepanov and Wackernagel (67). Transductants from BW2113 Δ*hchA*::Kan^r^ to BW2113Δ*yajL* were checked by PCR for transduction of the Δ*hchA*::Kan^r^ allele into the recipient strains using primer pairs DH492/493 (ext)-DH494/495 (int) and DH480/481 (ext)-DH482/483 (int), respectively. Plasmid constructions for expression of JCVI-Syn3A genes in E. coli are described in the Supplemental Methods at figshare (https://doi.org/10.6084/m9.figshare.20020574).

JCVI-Syn3A is a near-minimal bacterial cell first reported by Breuer et al. (3) that contains a subset of the genes in Mycoplasma mycoides subsp. *capri* strain GM12. Mycoplasmas were grown in SP4 broth (68) that contains 17% KnockOut serum replacement instead of 17% fetal bovine serum and is referred to as SP4-KO as described in the Supplemental Methods at figshare (https://doi.org/10.6084/m9.figshare.20020574). Construction of gene knockout mutants in JCVI-Syn3A was a multistep process, and two different protocols were used. These protocols are described in detail in the Supplemental data S2 file at figshare (https://doi.org/10.6084/m9.figshare.20020574).

### Mutation frequency assays for E. coli derivatives.

Overnight cultures in LB with added antibiotics and arabinose (0.02%) were diluted 100-fold under the same conditions and grown for another 24 h before dilutions were plated on LB and LB-rifampicin (25 μg/mL) to calculate a mutation ratio (number of colonies on Rif × dilution factor)/(number of colonies on LB × dilution factor).

### Protein expression and purification and enzyme assays.

All characterized JCVI-Syn3A-encoded proteins were expressed as His-tagged variants in E. coli and purified using Ni^2+^-NTA (nitrilotriacetic acid) columns as described in the Supplemental Methods at figshare (https://doi.org/10.6084/m9.figshare.20020574). *In vitro* activity assays for CoA disulfide reductase, for phosphatase with a range of substrates, NadD, glyoxalase, and deglycase are described in detail in the Supplemental Methods at the figshare URL above.

### Data availability.

The appendix and supplemental data have been deposited in the figshare data depository with the DOI https://doi.org/10.6084/m9.figshare.20020574.
